# Oral Herpes Zoster Infection Following COVID-19 Vaccination: A Report of Five Cases

**DOI:** 10.7759/cureus.19433

**Published:** 2021-11-10

**Authors:** Hiroshi Fukuoka, Nobuko Fukuoka, Toshiro Kibe, R. Shane Tubbs, Joe Iwanaga

**Affiliations:** 1 Oral Medicine, Fukuoka Dental Office, Satsuma, JPN; 2 Department of Oral and Maxillofacial Surgery, Field of Oral and Maxillofacial Surgery, Developmental Therapeutics Course, Graduate School of Medical and Dental Sciences, Kagoshima University, Kagoshima, JPN; 3 Department of Neurosurgery, Tulane Center for Clinical Neurosciences, Tulane University School of Medicine, New Orleans, USA; 4 Department of Neurosurgery and Ochsner Neuroscience Institute, Ochsner Health System, New Orleans, USA; 5 Department of Anatomical Sciences, St. George’s University, St. George’s, GRD; 6 Department of Neurology, Tulane University School of Medicine, New Orleans, USA; 7 Department of Anatomy, Dental and Oral Medical Center, Kurume University School of Medicine, Kurume, JPN

**Keywords:** dermatome, pandemic, palate, clinical anatomy, vaccination, covid-19, reactivation, infection, varicella zoster virus, oral herpes zoster

## Abstract

Recently, two cases of oral herpes zoster (HZ) following COVID-19 vaccines were reported. It was suggested that COVID-19 vaccine-related oral HZ cases might be missed or misdiagnosed as stomatitis or isolated oral herpes. In this report, five cases of oral HZ following COVID-19 vaccinations are presented. Four cases were observed on the hard palate (V2), and one case was found on the mandible (V3). Four patients did not receive any treatment for their oral HZ, but one patient also had skin reactions on her right orbit and ear and was thus treated with an antiviral drug. As these cases were seen during such a short period of time and in one practice, the relationship with the COVID-19 vaccination appears to be related.

## Introduction

It is generally accepted that varicella zoster virus (VZV) rests latent in the trigeminal ganglia (TG) and spinal dorsal root ganglia (DRG). The reactivation of the VZV in the TG causes herpes zoster (HZ) in the lower, middle, or upper face, or even in the oral cavity, depending on the involvement of the ophthalmic division (V1), the maxillary division (V2), or the mandibular division (V3) of the trigeminal nerve. The trigeminal nerve branches are involved in up to 20% of reported cases [1]. A recent study by Katz et al. concluded that the VZV reactivation is strongly associated with coronavirus disease 2019 (COVID-19) infection [2]. Although the mechanism of reactivation is still unclear, the immune system might also be affected by a COVID-19 infection. Since COVID-19 vaccines have been developed and used worldwide, skin reactions including HZ by reactivation of VZV following COVID-19 vaccines have been reported in the literature [3-5].

Recently, Iwanaga et al. reported two cases of oral HZ along the V2 dermatome following administration of COVID-19 vaccines and suggested that COVID-19 vaccine-related oral HZ cases might be missed or misdiagnosed as stomatitis or isolated oral herpes [6]. Thus, oral HZ needs to be given more attention during intraoral examinations, especially by dentists treating patients with a history of COVID-19 vaccination. Here, five cases of oral HZ following COVID-19 vaccinations are presented.

## Case presentation

**Figure 1:**
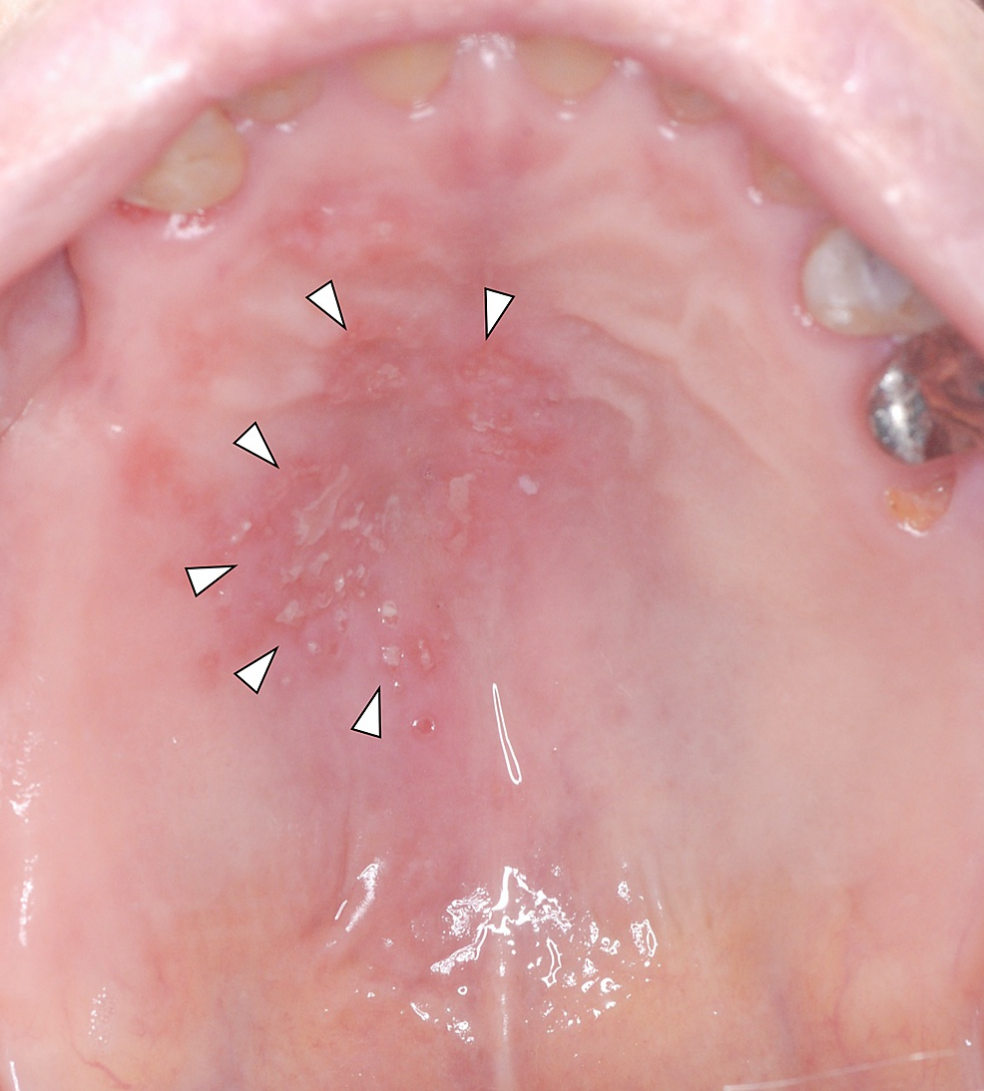
A mirror image of the multiple unilateral herpes zoster on the left hard palate (arrowheads)

Since individuals in the town of Satsuma (Japan) began receiving COVID-19 vaccinations in May 2021, five Japanese patients (four females and one male), whose age range was 59-97 years (mean age: 74.6 years), visited the authors’ (H.F. and N.F.) general dental practice for multiple unilateral small vesicles and ulcers on their hard palate (V2) and mandible (V3) through October 2021 (Figure 1).

The legions were all diagnosed as oral HZ based on clinical findings (unilateral acute rash for less than seven days with or without pain) [7]. All five patients received the BNT162b2 (Pfizer) vaccine one to three weeks before the first visit to the dental practice, as BNT162b2 (Pfizer) was the only type of vaccination available in Satsuma. The patients’ previous history of HZ, varicella, or VZV vaccination was unknown. Four patients did not receive any treatment for their oral HZ, but one patient (case 2) also had a skin reaction on her right orbit and ear and was treated with an antiviral drug by her doctor (Table 1). The authors had been practicing for 10 years and had seen the oral HZ patients a couple of times per year in their practice before May 2021.

**Table 1:** Five cases of oral HZ following the COVID-19 vaccination HZ, herpes zoster

Case	Age (years)	Sex	Latent period of vaccine-related HZ	Affected area (dermatome)	Vaccinated side (arm)	Vaccine type	Dose reactivating HZ	Medical history	HZ in other areas
1	63	F	2 weeks	Left palate (V2)	Left	BNT162b2	First dose	Asthma	None
2	70	F	3 weeks	Right mandible (V3)	Right	BNT162b2	Second dose	Hypertension	Right orbit and ear
3	84	F	1 week	Right palate (V2)	Left	BNT162b2	Second dose	Arrhythmia, stroke, Ménière's disease	None
4	97	F	3 weeks	Left palate (V2)	Left	BNT162b2	First dose	Hypertension, stroke, dementia	None
5	59	M	3 weeks	Left palate (V2)	Left	BNT162b2	Second dose	None	None

## Discussion

HZ with or following COVID-19

A variety of dermatological signs and symptoms have been reported worldwide following COVID-19 infection. HZ is one of these dermatological signs [8,9]. Some patients had reactivation of VZV following recovery from symptomatic COVID-19 [10,11].

Vaccine-related reactivation of VZV after COVID-19 vaccination

It is generally accepted that vaccines for viruses such as rabies, influenza, hepatitis A, and Japanese encephalitis can trigger VZV reactivation [12-15]. The number of reported cases of COVID-19 vaccine-related HZ is increasing as the number of individuals receiving the vaccine is increasing. However, in regard to the time following vaccination until the development of HZ, the definition of “vaccine-related HZ” is still not defined. Some authors have considered one week after vaccination [16], and others have used three weeks to determine if the HZ was related to vaccine [16,17]. The previous review by Iwanaga et al. found that the reported latent period of vaccine-related HZ ranged from one to 24 days following COVID-19 vaccination [6]. In this report, the latent period of vaccine-related HZ ranged from one to three weeks after either the first or second dose.

Oral HZ following COVID-19 vaccination

The ophthalmic division (V1) of the trigeminal nerve is the most commonly involved division with HZ regarding the maxillofacial manifestations, and less so in the maxillary (V2) and mandibular (V3) divisions [18]. In four out of five cases in the present study, the HZ was observed on the hard palate (V2) (one was on the mandible [V3]). With these four cases and the two earlier cases reported by us, it seems that the hard palate (V2), specifically the greater palatine nerve or nasopalatine nerve, might be more commonly affected by HZ compared to the mandible (V3). The patients usually complain of severe pain and a rash in the corresponding area. Interestingly, although oral HZ is a well-known finding caused by reactivation of VZV, any oral HZ cases reactivated after COVID-19 vaccination were not identified in a PubMed search conducted in August 2021 by Iwanaga et al. [6]. These authors presented two cases of oral HZ (both were on the hard palate) with a latent period of 11 and 38 days, respectively, following COVID-19 vaccinations. They concluded that many cases of oral HZ might have been missed or misdiagnosed, as one general practice had already experienced two oral HZ cases during a short period of time [6]. We agreed with this notion, as the five cases presented occurred during a short period of time (i.e., five months). Other concurrent symptoms, such as those found in case 2 (V1) of the present study, should be noted to find related legions as, for example, the oral HZ in V2 might appear with a midfacial symptom, which is also innervated by V2 [18]. The HZ is relatively easy to diagnose based on clinical findings such as a unilateral acute rash present for less than seven days with or without pain [7]. It can consist of multiple legions. When findings of HZ are found on a patient’s skin, the oral cavity should also be examined, and vice versa. For example, case 2 in this study presented with HZ on the right mandible, and the patient’s history revealed that there was also concurrent HZ on the ipsilateral orbit and ear. Thus, oral HZ might be found by examining more closely a patient’s history of COVID-19 vaccination and other concurrent cases of HZ.

An increasing incidence of HZ infection in general has also been reported in the literature. In the United States, 0.76 per 1,000 people had an HZ infection between 1945 and 1949, whereas 3.15 per 1,000 people had an HZ infection between 2000 and 2007 [19]. COVID-19 infection and COVID-19 vaccination can be other triggers that increase oral HZ patients. Although the patients’ previous history of HZ, varicella, or VZV vaccination was unknown in the present study, some authors reported that even patients who had a history of VZV vaccination had COVID-19 vaccine-related HZ [6]. Therefore, any patient who has had COVID-19 vaccinations can be affected by oral HZ.

## Conclusions

We presented five cases of COVID-19 vaccine-related oral HZ. Four cases presented with oral HZ in the V2 dermatome and one in the V3 dermatome. Together with our previously reported cases, COVID-19 vaccine-related oral HZ might more often involve the V2 dermatome. As these cases were seen during such a short period of time and were all from a single practice, additional cases will most likely be reported over time. The oral cavity in a patient who has a history of COVID-19 vaccination should be examined regardless of the symptomatic skin findings of HZ. The authors hope their cases will serve as a start to future studies and cases of oral HZ following COVID-19 vaccination.
